# Very low-calorie ketogenic diet (VLCKD) in the management of hidradenitis suppurativa (*Acne Inversa*): an effective and safe tool for improvement of the clinical severity of disease. Results of a pilot study

**DOI:** 10.1186/s12967-024-04853-0

**Published:** 2024-02-13

**Authors:** Ludovica Verde, Sara Cacciapuoti, Giuseppina Caiazzo, Matteo Megna, Fabrizio Martora, Annarita Cavaliere, Maria Mattera, Maria Maisto, Gian Carlo Tenore, Annamaria Colao, Silvia Savastano, Giovanna Muscogiuri, Luigi Barrea

**Affiliations:** 1 Department of Public Health, University of Naples Federico II, Via Sergio Pansini 5, 80131 Naples, Italy; 2 Centro Italiano per la cura e il Benessere del Paziente con Obesità (C.I.B.O), Unità di Endocrinologia, Diabetologia e Andrologia, Dipartimento di Medicina Clinica e Chirurgia, Università degli Studi di Napoli Federico II, Via Sergio Pansini 5, 80131 Naples, Italy; 3 Department of Clinical Medicine and Surgery, Section of Dermatology, University of Naples Federico II, Naples, Italy; 4 Department of Advanced Biomedical Sciences, University of Naples Federico II, Naples, Italy; 5 ChimNutra labs, Department of Pharmacy, University of Naples Federico II, via Domenico Montesano 49, 80131 Naples, Italy; 6 Unità di Endocrinologia, Diabetologia e Andrologia, Dipartimento di Medicina Clinica e Chirurgia, Università degli Studi di Napoli Federico II, Via Sergio Pansini 5, 80131 Naples, Italy; 7 Cattedra Unesco “Educazione Alla Salute E Allo Sviluppo Sostenibile”, University Federico II, Naples, Italy; 8 Dipartimento di Scienze Umanistiche, Università Telematica Pegaso, Centro Direzionale, Via Porzio, Isola F2, 80143 Naples, Italy

**Keywords:** Hidradenitis suppurativa, Acne inversa, Very Low-Calorie Ketogenic Diet, VLCKD, Ketogenic diet, Obesity, TMAO, Inflammation, Diet, Nutrition, Phase angle, Skin

## Abstract

**Background:**

Hidradenitis suppurativa (HS), an inflammatory-based dermatological condition often associated with obesity, poses significant challenges in management. The very low-calorie ketogenic diet (VLCKD) has shown efficacy in addressing obesity, related metabolic disorders, and reducing chronic inflammation. However, its effects on HS remain underexplored. In this prospective pilot study, we aimed to investigate the impact of a 28-day active phase of VLCKD on HS in a sample of treatment-naive women with HS and excess weight.

**Methods:**

Twelve women with HS and overweight or obesity (BMI 27.03 to 50.14 kg/m^2^), aged 21 to 54 years, meeting inclusion/exclusion criteria and agreeing to adhere to VLCKD, were included. Baseline lifestyle habits were assessed. The Sartorius score was used to evaluate the clinical severity of HS. Anthropometric parameters (waist circumference, weight, height, and body mass index), body composition *via* bioelectrical impedance analysis, levels of trimethylamine N-oxide (TMAO), oxidized low-density lipoprotein (oxLDL), and derivatives of reactive oxygen metabolites (dROMs) were assessed at baseline and after 28 days of the active phase of VLCKD.

**Results:**

VLCKD led to general improvements in anthropometric parameters and body composition. Notably, a significant reduction in the Sartorius score was observed after the intervention (Δ%: − 24.37 ± 16.64, p < 0.001). This reduction coincided with significant decreases in TMAO (p < 0.001), dROMs (p = 0.001), and oxLDL (p < 0.001) levels. Changes in the Sartorius score exhibited positive correlations with changes in TMAO (p < 0.001), dROMs (p < 0.001), and oxLDL (p = 0.002).

**Conclusion:**

The 28-day active phase of VLCKD demonstrated notable improvements in HS severity and associated metabolic markers, highlighting the potential utility of VLCKD in managing HS and its association with metabolic derangements in women with overweight or obesity.

**Supplementary Information:**

The online version contains supplementary material available at 10.1186/s12967-024-04853-0.

## Introduction

Hidradenitis suppurativa (HS), also referred to as *acne inversa*, is a chronic inflammatory skin condition that primarily affects body areas with intertriginous sites and a high density of apocrine glands, such as the axillae, inframammary folds, and anogenital regions [1]. This condition typically emerges during adolescence and young adulthood and can persist throughout a person's life. HS is characterized by recurring inflammatory nodules and, in more severe cases, the development of abscesses accompanied by sinus tracts and extensive scarring. This leads to distressing symptoms, including pain, malodor, and physical disfigurement, resulting in significant psychological strain [1]. The prevalence of HS is likely underreported; however, current data estimate a global prevalence of 0.00033–4.1% [2]. Recent evidence has shifted away from apocrine glands as the primary origin of HS, highlighting defects in the follicular portion of the folliculopilosebaceous units as a key contributor to the disease's pathogenesis [3].

Patients with HS exhibit a higher obesity prevalence compared to the general population. In 2008, two case–control studies involving 67 and 302 patients with HS [4], respectively, highlighted these associations. In the first study, 16.4% of patients with HS suffered from obesity (BMI ≥ 30.0 kg/m^2^) versus 13.3% of controls. Overweight (BMI 25.0–29.0 kg/m^2^) was 26.9% in patients with HS versus 20.4% in controls. The second study reported 21.4% obesity in patients with HS versus 17.1% controls, with overweight percentages at 21.8% (HS) and 8.5% (controls). Cross-sectional design limits establishing causation. Other studies note that over 75.0% of patients with HS experience overweight or obesity [5, 6], correlating disease severity with a higher BMI [7–9].

The hormonal changes associated with obesity, androgen access, and dietary effects likely exacerbate HS, as do the larger areas of skin folds, sweat retention, and friction [10]. In contrast, exercise can be painful for HS patients, contributing to the vicious cycle of obesity and HS. The underlying inflammatory process is likely influenced by the increased levels of proinflammatory cytokines seen in patients with obesity [10]. Interestingly, a weight loss of 15% has been shown to significantly reduce HS severity [11].

However, several other factors may contribute to the development and clinical severity of HS, including altered microbial composition (microbiota dybiosis) and nutrition [12, 13]. In this context, we previously provided the first evidence that circulating levels of Trimethylamine N-Oxide (TMAO), a gut-derived metabolite associated with inflammation and cardiometabolic risk, were increased in patients with HS and were associated with the clinical severity of the disease [14].

Treatment of HS has been challenging, as it does not respond reliably to medical therapies. Given limited treatment options, dietary modifications have gained considerable interest as a possible treatment option for HS [15]. Although management through diet and lifestyle modifications is a primary interest of the patients with HS community, there is a lack of consensus on recommendations due to the paucity of evidence. In this regard, the British Association of Dermatologists noted in their 2018 guidelines that there were no high-quality studies supporting the beneficial effect of diet in HS [16]. The United States and Canadian Hidradenitis Suppurativa Foundations also published clinical management guidelines in 2019, concluding that there wasn't enough evidence to support the routine use of vitamin D, zinc, and dairy avoidance (recommendations classified as class C or level II/III evidence) [17]. Despite the interest in dietary modifications among the patients in the HS community, the lack of evidence has made it challenging to establish clear guidelines for its management.

Finally, among the exacerbating factors in HS, diet plays a key role [18]. Notably, recent studies have highlighted the potential impact of diet on HS, including the significance of the Mediterranean diet [19, 20]. However, there remains much to be studied in the pursuit of establishing the role of diet on disease outcomes. Dietary interventions should always be considered in addition to pharmacological therapy. Recently proposed as an obesity management nutritional strategy, the Very Low-Calorie Ketogenic Diet (VLCKD), through the production of ketone bodies, was associated with a significant reduction in body weight, inflammatory status, and gut microbiota composition [21–23]. While data on the efficacy of VLCKD in psoriasis is available, the efficacy of VLCKD in improving the clinical severity of HS has not been established in clinical studies, and there is no evidence in the literature [15, 18]. Dietary strategies for reducing inflammation and body weight represent a topic of great interest to both nutritionists and dermatologists. Recent research [24, 25] suggests a compelling anti-inflammatory role for VLCKD, potentially surpassing low-fat diets [26]. Although a comprehensive meta-analysis is lacking, emerging evidence highlights VLCKD's notable anti-inflammatory effects [26]. Thus, as HS is an inflammatory-based disease and often associated with obesity, in this prospective pilot study we aimed to investigate the effects of 28 days of the active phase of VLCKD in a sample of naive-treatment women with HS.

## Methods

### Design and setting

This prospective pilot study was conducted on women with HS at the Dermatology Unit of the Department of Clinical Medicine and Surgery of the University Federico II of Naples. The study ran from September 2021 to July 2023. Ethical authorization for the study was granted by the Local Ethics Committee (reference no. 50/20), and all procedures were conducted in strict compliance with the World Medical Association's Code of Ethics, in particular the Declaration of Helsinki, which defines the principles of human experimentation. The objectives and procedures of the study were fully communicated to all participants, ensuring complete clarity. Prior to involvement, written informed consent was obtained from each participant stating their willingness to take part in the study.

### Population study

In order to enhance the uniformity of the patient groups, our attention was directed solely towards Caucasian women who had excess weight or obesity and were afflicted by HS. These women were specifically drawn from the geographical vicinity surrounding the Naples metropolitan area in Campania, Italy. Comprehensive medical information was collected from all women. The inclusion criteria for participation in the study were as follows:

- Treatment-naive subjects;
- BMI ≥ 25.0 kg/m^2^;
- Fulfillment of all three diagnostic criteria for HS: presence of characteristic lesions, involvement of anatomical sites in typical regions, and an ongoing disease course marked by relapses and chronicity;
- Women who had not previously received treatment for HS;
- HS diagnosis was established at least 6 months before the study, with no medical therapy for a minimum of 3 months.

Women were excluded if they met any of the following exclusion criteria:

- Occasional or current use of systemic treatments (such as anti-inflammatory, anti-obesity, biologics, cyclosporine A, rifampicin–moxifloxacin–metronidazole, clindamycin–rifampicin, dapsone, ertapenem, tetracycline, acitretin, and isotretinoin) or other medications for HS, including topical antibiotics;
- Suffered from any other active skin condition (like psoriasis or acne) that might interfere with HS assessment;
- Had one or more contraindications for VLCKD according to current guidelines from the European Association for the Study of Obesity (EASO);
- Displayed symptoms or signs indicative of androgen excess or underlying endocrine disorders;
- Adherence to hypocaloric diets, specific dietary patterns (including vegetarian or ketogenic diets), or use of antioxidants, vitamins, minerals, or probiotics within the past three months;
- Presence of clinical conditions or use of medications affecting fluid balance, such as liver or renal failure, cancer, and other chronic or acute illnesses as determined by comprehensive medical examinations and laboratory tests;
- Possess implanted pacemakers or defibrillators, considering the potential theoretical interference with the activity of the bioelectrical impedance analysis (BIA) device;
- Had a history of clinical conditions that, in the judgment of the Dermatologist, Endocrinologist and Nutritionist could put the patient at risk if they participated in this study.

**Fig. 1:**
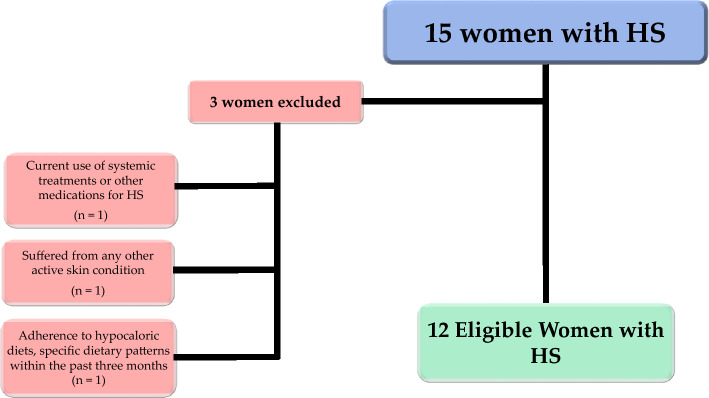
Flow chart of study participants. HS, hidradenitis suppurativa; BMI, body mass index

Figure 1 shows the flow chart of women included in the analysis.

### Study protocol

**Fig. 2:**
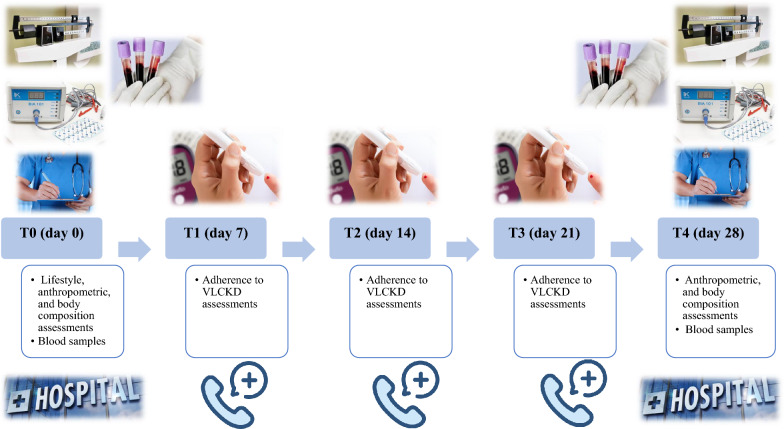
Study protocol. VLCKD, very low-calorie ketogenic diet

The study protocol encompassed a series of five visits (T0, T1, T2, T3, T4), each occurring every 7 days over a total span of 28 days, constituting the active phase of VLCKD, as depicted in Fig. 2. At baseline (T0), a comprehensive assessment carried out by a team of Dermatologist, Endocrinologist, and Nutritionist was conducted to ascertain the eligibility of women. Those meeting the criteria for inclusion and exclusion were enrolled in the study and provided their written informed consent. At this point, a Nutritionist undertook lifestyle, anthropometric, and body composition assessments. Women were then provided with personalized instructions for adhering to the VLCKD. This included receiving individualized dietary plans and scheduled replacement meals for the week. Simultaneously, with the support of nursing staff, blood samples were collected for general biochemical tests, oxidative stress evaluation, and TMAO levels. Finally, women were advised to maintain their existing level of physical activity throughout the study duration. During the subsequent follow-up visits (T1, T2, and T3), women underwent assessments of adherence to VLCKD by the Nutritionist via telephone interview. Adherence was gauged through ketone body measurements extracted from capillary blood samples, and the Nutritionist recorded whether the patient exhibited ketosis (YES/NO). The Nutritionist also documented any deviations in physical activity levels or deviations from the food and beverage consumption patterns outlined in the VLCKD protocol. In the last visit (T4, day 28), a final round of dermatological, endocrinological, and nutritional assessments was conducted in the presence of the doctors (Dermatologist and Endocrinologist) and the Nutritionist. Blood samples were collected once more for the repetition of biochemical, oxidative stress, and TMAO analyses.

### Hidradenitis suppurativa assessment

Since there is no established benchmark for this purpose, the evaluation of HS was conducted using the Sartorius HS score [1]. The Sartorius HS score is a clinical classification system that involves the enumeration of individual fistulas and nodules in seven anatomical regions. It also entails measuring the greatest distance between two similar lesions within each of these anatomical areas, namely the axilla, gluteal, groin, genital, and other inflammatory sites on the left and/or right sides [9]. The clinical severity of HS was determined by two unbiased Dermatologists who were unaware of the study's design, thus minimizing any potential biases. In addition, we evaluated the Dermatology Life Quality Index (DLQI), a standardized instrument used to assess the impact of dermatitis and other skin conditions on patients' quality of life. It is a self-assessment questionnaire that measures different aspects of daily life influenced by the presence of dermatological symptoms. The DLQI consists of ten questions divided into six categories: symptoms and feelings, embarrassment, daily activities, clothing, leisure, and interpersonal relationships. Each question is rated on a four-point scale, with scores ranging from 0 to 3, representing 'no impact', 'minor impact', 'moderate impact' and 'major impact', respectively. To calculate the total DLQI score, the scores obtained from the ten questions are added together, with a total possible score ranging from 0 (no impact on quality of life) to 30 (maximum impact on quality of life).

### Lifestyle habits

As previously reported [27–29], we defined current smokers as women smoking at least one cigarette *per* day, former smokers as women who had stopped smoking at least one year before the interview, and non-current smokers as the remaining women. Women habitually engaging in at least 30 min *per* day of aerobic exercise (YES/NO) were defined as physically active.

### Anthropometric assessment

The same Nutritionist performed the anthropometric assessment. All women were assessed between 8 and 10 a.m. after an overnight fast, wearing light clothing and no shoes. Body weight was measured to the nearest 0.1 kg using a calibrated balance beam scale, and height was measured to the nearest 0.5 cm using a wall-mounted stadiometer. BMI was calculated by dividing weight (in kg) by height squared (in meters). BMI was classified according to the World Health Organization's criteria for normal weight, overweight, and I, II and III grades of obesity. Waist circumference (WC) was measured to the nearest 0.1 cm with a no-stretch tape measure at the natural indentation or halfway between the lower edge of the rib cage and the iliac crest if no natural indentation was visible.

### Bioelectrical impedance analysis

Body composition was evaluated using a BIA phase-sensitive system, administered by an experienced nutritionist, as previously documented [22, 30–32]. The system applied an 800-µA current at a signal frequency of 50 kHz, using the BIA 101 RJL instrument from Akern Bioresearch in Florence, Italy. The examination was conducted in accordance with the guidelines provided by the European Society of Parenteral and Enteral Nutrition (ESPEN) [33]. Electrodes were positioned on the hand and the corresponding foot, following the protocol established by Kushner in 1992 [34]. The PhA was calculated based on conditions under 50 kHz using the following formula: PhA (°, degrees) = arctangent of reactance (Xc) divided by resistance (R) multiplied by (180/π).

### VLCKD intervention

According to EASO guidelines, VLCKD consists of three phases (active, re-education, and maintenance) [35]. The active phase of VLCKD was collaboratively devised by a Nutritionist and endorsed by an Endocrinologist. The dietary composition adhered to specific parameters, with a total energy intake of less than 800 kcal *per* day. This energy was derived from a distribution of 13% from carbohydrates (less than 30 g *per* day), 43% from protein (1.3 g *per* kilogram of ideal body weight), and 44% from fat. The ideal body weight (kg) was calculated using the Lorentz equation: ideal body weight = height (cm) – 100 − [(height − 150)/2] [36]. Throughout VLCKD, meals with high biological value were provided as replacements, and the protein content originated from sources such as whey, soy, eggs, and peas. To ensure nutritional adequacy during the VLCKD, supplementation was introduced. This included B-complex vitamins, vitamins C and E, essential minerals like potassium, sodium, magnesium, and calcium, as well as omega-3 fatty acids. An example of a VLCKD with meal replacements is reported in Additional file 1.

### Biochemical assessment

Blood samples were obtained through venipuncture between 8 and 10 in the morning, following an overnight period of fasting, with the procedure conducted by the nursing staff. Subsequently, the samples were transported to the local laboratory and processed in accordance with the established local standards and protocols. The measurements included assessments of glucose and lipid profiles, electrolyte levels, uric acid concentrations, liver enzyme activities, and indicators of kidney function. The intra- and inter-assay CVs were below 3% for all the samples. Additionally, the Homeostatic Model Assessment for Insulin Resistance (HoMA-IR) was computed for each woman using the formula [fasting glucose (mmol/l) × fasting insulin (mU/ml)/22.5] [37]; the intra-assay CV for insulin was < 5.5%.

### Evaluation of the oxidized low-density lipoprotein levels

Blood samples were obtained using the brachial puncture method and placed into heparin vacuum tubes with a 5-mL capacity. After being centrifuged at 3000 rpm for 10 min at room temperature, the sera were isolated and stored at a temperature of − 80 °C for subsequent analysis within a maximum period of 6 months. The measurement of plasma levels of oxidized low-density lipoprotein (ox-LDL) was conducted through the employment of the LP-CHOLOX test on an automated analyzer known as Free Carpe Diem, provided by Diacron International, Grosseto, Italy. This test employed a commercial kit from the same manufacturer and was executed following the provided instructions. The LP-CHOLOX test assesses a category of hydroperoxides originating from lipid peroxidation, primarily comprising oxidized cholesterol. These hydroperoxides have the capability to induce the conversion of ferrous iron (Fe^2+^) to ferric iron (Fe^3+^), thereby promoting oxidation. The LP-CHOLOX test is reliant on a spectrophotometric assessment conducted at a wavelength of 505 nm, measuring the development of a colored complex formed by the interaction of Fe3 + and thiocyanate. The resulting absorbance values exhibit a direct correlation to the concentration of lipoperoxides present, and these values are standardized against a specific solution (400 μEq/L). For all the analyzed samples, the calculated intra- and inter-assay CV% values were below t2.8% indicating the high reliability and precision of the used method. The outcomes are expressed in μEq/L units, with reference ranges established as follows: normal (≤ 599 μEq/L), minor alteration (600 to 799 μEq/L), moderate alteration (800 to 999 μEq/L), and significant alteration (≥ 1000 μEq/L) [19].

### Evaluation of dROMS

The assessment of oxidant ability in the plasma was conducted using the d-ROM Lab test developed by Innovatics Laboratories Inc. This analytical method indirectly gauges the presence of organic hydroperoxides (ROOH) in a sample, which are the primary contributors to oxidant ability. The methodology is centered around the oxidation of iron (or copper) through Fenton's reaction. The resulting change in color, induced by the addition of the oxidizable chromogen substrate N,N-diethyl-paraphenylendiamine, is quantified through photometric measurements. The outcomes are reported in "Carratelli units" (U Carr), with 1 U Carr being equivalent to 0.08 mg of hydrogen peroxide (H_2_O_2_) per 100 mL [38]. The reliability of the performed analysis was assessed by the calculation of CV% at the intra- and inter-assay levels for all collected samples, which resulted in an estimated CV% below 3.72% for both parameters.

### Trimethylamine-N-oxide assessment

Blood samples aimed at determining TMAO levels in the serum were stored at a temperature of -80° C. This preservation approach was selected due to evidence indicating that TMAO remains stable over extended durations under these specific conditions [39]. The quantification of TMAO levels in the serum was conducted using the procedure delinelated by Beale and Airs [40], which was detailed in our previous studies [14, 41]. For chromatographic separation, a guard column (HILIC) was employed in conjunction with a Luna HILIC column (measuring 150 mm × 3 mm, with 5 µm particles), both provided by Phenomenex (located in Torrance, CA, USA). The sensitivity of the analytical method was described by the determination of a Limit of Detection (LOD) of 2 ng/ml and a Limit of Quantification (LOQ) of 6 ng/ml. In order to evaluate the precision of the method used, the CV% at intra- and inter-day levels were calculated at three different TMAO levels (0.3, 3, and 13 µM), resulting in a calculated intra-day CV% of 8.12, 1.54, and 1.52 µM and of an inter-day CV% of 9.2, 2.2, and 3.3 µM, respectively. Similarly, over the same TMAO levels, the accuracy of the method was calculated by the evaluation of the accuracy (% bias) both intraday and intraday, leading to an estimation of % bias ranging from − 3.52 to 0.66, indicating the high reliability of the used LC/MS method.

### Statistical Analysis

G* Power was used to conduct an a priori sample size calculation. In the absence of preliminary data on the efficacy of this intervention and considering an effect size of 0.8 (clinically meaningful, according to Cohen), the sample size sufficient to have a power of 80% with a 1-tailed type-I error of 5% was 12 patients. The data analysis was conducted using the MedCalc^®^ package (Version 12.3.0, 1993–2012 MedCalc Software bvba—MedCalc Software, Mariakerke, Belgium) and IBM SPSS Statistics Software (PASW Version 21.0, SPSS Inc., Chicago, IL, USA). The statistical analysis included only women who had measurements at both the baseline and after 28 days of the active phase of VLCKD. Results were presented in the form of mean ± standard deviation (SD) for continuous variables and as a number and percentage (n, %) for categorical variables. To assess data distribution, the Kolmogorov–Smirnov test was employed. The differences between baseline and measurements after 28 days of the active phase of VLCKD were compared using the paired Student’s *t*-test. Spearman’s correlation was utilized to investigate the association between baseline and measurements after 28 days of the active VLCKD phase in terms of percentage changes (delta ∆%). Furthermore, a multiple linear regression analysis model employing the stepwise method used the Δ% Sartorius score as a dependent variable to estimate the predictive value of changes in PhA, TMAO, dROMs, and ox-LDL after 28 days of the active phase of VLCKD, expressed as R^2^, beta (*β*), and *t*.

## Results

The average ideal weight calculated (Lorentz equation) in the study population was 59.21 ± 4.48 kg. Based on ideal body weight, the protein intake of the VLCKD was calculated (1.3 g of protein *per* ideal body weight). The average protein content of the diet was 76.97 ± 5.82 g.

The study population included 12 women participants with overweight or obesity (BMI 27.03 to 50.14 kg/m^2^), aged 21 to 54 years. The preponderance of women was characterized as sedentary (83.3%) and non-smokers (58.3%). None of the patients changed their physical activity levels or cigarette smoking habits during the 28 days of treatment. Furthermore, of interest, no patient reported adverse effects important to suspending the VLCKD protocol, as stated during the three telephone interviews. All patients also declared the presence of a state of ketosis.

Anthropometric characteristics and body composition of the study population at baseline and after 28 days of the active phase of VLCKD are reported in Table 1. After 28 days of the active phase of the VLCKD, in the entire study population, both BMI (Δ%: − 7.94 ± 1.95, p < 0.001) and WC (Δ%: − 7.56 ± 6.34, p = 0.002) were significantly reduced compared to baseline. After 28 days of the active phase of VLCKD, FM (%) (Δ%: − 14.26 ± 14.32, p = 0.004) was significantly reduced while FFM (%) (Δ%: 10.72 ± 9.87, p = 0.004) slightly increased. A significant increase in PhA (Δ%: 15.54 ± 10.43, p < 0.001) compared to the baseline was also detected.

**Table 1:** Anthropometric characteristics and body composition of the study population at baseline and after 28 days of the active phase of VLCKD

Parameters (N = 12)	Baseline	Day 28 of VLCKD	p-value	Δ %
Anthropometric parameters				
Weight (kg)	101.43 ± 14.52	93.51 ± 14.77	** < 0.001**	− 7.94 ± 1.95
BMI (kg/m^2^)	35.95 ± 5.89	33.15 ± 5.88	** < 0.001**	− 7.94 ± 1.95
WC (cm)	109.5 ± 17.34	100.92 ± 15.66	**0.002**	− 7.56 ± 6.34
BIA parameters				
R (Ω)	493.25 ± 46.59	469.67 ± 40.30	**0.003**	− 4.61 ± 4.04
Xc (Ω)	50.83 ± 4.59	56.25 ± 7.46	**0.002**	10.45 ± 9.28
Na/K	0.87 ± 0.14	0.86 ± 0.14	0.754	− 0.51 ± 9.91
BCM (kg)	29.97 ± 4.01	32.83 ± 3.68	**0.026**	10.56 ± 14.26
TBW (%)	41.50 ± 5.16	45.76 ± 7.04	**0.005**	10.25 ± 9.92
ECW (%)	46.26 ± 3.58	42.34 ± 3.43	** < 0.001**	− 8.35 ± 5.12
ICW (%)	53.74 ± 3.58	57.66 ± 3.43	** < 0.001**	7.41 ± 4.59
FM (kg)	45.19 ± 13.24	36.49 ± 14.77	** < 0.001**	− 21.01 ± 13.80
FM (%)	43.89 ± 6.59	37.85 ± 9.38	**0.004**	− 14.26 ± 14.32
FFM (kg)	56.23 ± 4.99	57.02 ± 3.66	0.580	1.90 ± 8.90
FFM (%)	56.12 ± 6.59	62.15 ± 9.38	**0.004**	10.72 ± 9.87
MM (kg)	25.96 ± 3.81	27.13 ± 2.64	0.268	5.95 ± 15.02
MM (%)	25.96 ± 4.70	29.76 ± 5.92	**0.009**	15.25 ± 16.56
BCMI	10.63 ± 1.60	11.56 ± 1.37	**0.001**	9.48 ± 7.92
PhA (°)	5.93 ± 0.77	6.83 ± 0.80	** < 0.001**	15.54 ± 10.43

A *p*–value in bold type denotes a significant difference (p < 0.05). VLCKD, very low-calorie ketogenic diet; Δ%, percentage change; *BMI* body mass index, *WC* waist circumference, *BIA* body impedance analysis, *R* resistance, *Xc* reactance, *BCM* body cell mass, *TBW* total body water, *ECW* extracellular water, *ICW* intracellular water, *FM* fat mass, *FFM* fat free mass, *MM* muscle mass, *BCMI* body cell mass index, *PhA* phase angle

Biochemical parameters of the study population at baseline and after 28 days of the active phase of VLCKD are reported in Table 2. Noticeable shifts were evident in the lipid profile. In particular, at the end of the 28-day active phase of the VLCKD total (Δ %: -19.41 ± 15.19, p = 0.002), HDL cholesterol (Δ %: − 14.02 ± 12.23, p = 0.002) and LDL cholesterol (Δ %: − 23.16 ± 18.82, p = 0.002) decreased significantly compared to baseline.

**Table 2:** Biochemical parameters of the study population at baseline and after 28 days of the active phase of VLCKD

Parameters (N = 12)	Baseline	Day 28 of VLCKD	p-value	Δ %
Fasting plasma glucose (mg/dL)	88.82 ± 7.95	80.64 ± 8.76	0.084	− 8.15 ± 15.73
Insulin (μU/mL)	12.48 ± 6.68	9.88 ± 6.60	0.218	− 16.70 ± 47.24
HoMA− IR	2.71 ± 1.50	2.09 ± 1.55	0.248	− 17.93 ± 55.15
Total cholesterol (mg/dL)	184.73 ± 26.01	147.55 ± 30.62	**0.002**	− 19.41 ± 15.19
HDL cholesterol (mg/dL)	61.22 ± 11.37	53.04 ± 13.86	**0.002**	− 14.02 ± 12.23
LDL cholesterol (mg/dL)	103.38 ± 24.50	77.13 ± 20.05	**0.003**	− 23.16 ± 18.82
Triglycerides (mg/dL)	102.36 ± 57.58	82.82 ± 32.12	0.223	− 7.77 ± 32.07
Azotemia (mg/dL)	32.00 ± 9.38	28.50 ± 9.95	0.158	− 9.33 ± 21.96
Creatinemia (mg/dL)	0.76 ± 0.17	0.75 ± 0.12	0.777	0.59 ± 14.50
Uricemia (mg/dL)	4.93 ± 0.92	5.39 ± 1.13	0.197	10.58 ± 22.13
AST (U/L)	25.50 ± 10.82	24.17 ± 10.67	0.258	− 3.80 ± 15.68
ALT (U/L)	29.67 ± 19.13	25.58 ± 18.34	0.055	− 11.68 ± 21.10
γGT (U/L)	15.92 ± 4.72	13.92 ± 4.25	0.074	− 10.60 ± 21.48
ESR (mm/h)	28.44 ± 15.21	22.44 ± 18.25	0.307	− 18.30 ± 47.46
Sodiemia (mmol/L)	4.30 ± 0.27	4.33 ± 0.37	0.591	0.31 ± 1.89
Potassiema (mmol/L)	4.30 ± 0.27	4.33 ± 0–37	0.700	0.44 ± 4.49

A p-value in bold type denotes a significant difference (p < 0.05). VLCKD, very low-calorie ketogenic diet; Δ%, percentage change; HoMA-IR, homeostatic model for assessment of insulin resistance; *HDL*, high density lipoprotein, *LDL* low density lipoprotein, *AST* aspartate transaminase, *ALT* alanine transaminase, *γGT* γglutamyltransferase, *ESR* erythrocyte sedimentation rate

**Fig. 3:**
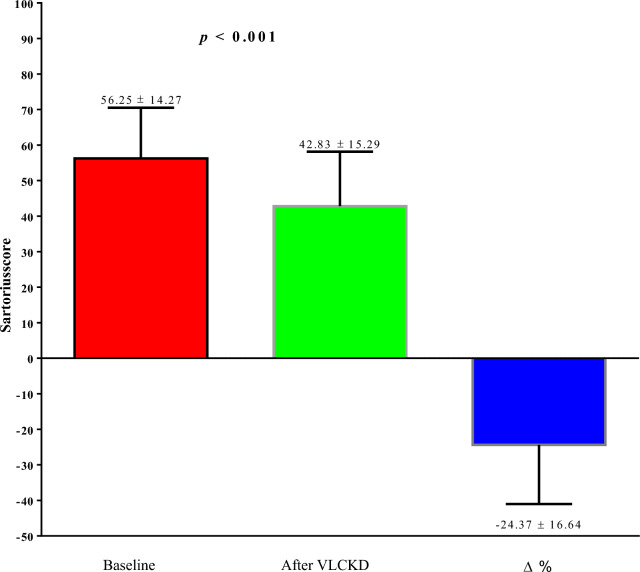
Sartorius score of the study population at baseline and after 28 days of the active phase of VLCKD. VLCKD, very low-calorie ketogenic diet; Δ%, percentage change

Of note, after 28 days of the active phase of VLCKD, both the DLQI score (Δ%: − 44.62 ± 35.74, p = 0.001), and the Sartorius score (Δ%: -24.37 ± 16.64, p < 0.001. Figure 3) decreased significantly compared to baseline.

Inflammation, oxidative stress, and dysbiosis parameters in the study population at baseline and after 28 days of the active phase of VLCKD are reported in Table 3. After 28 days of the active phase of VLCKD, in the entire study population, we observed significant reductions in ox-LDL (Δ%: − 25.53 ± 7.64, p < 0.001), dROMs (Δ%: − 21.10 ± 12.86, p = 0.001), and TMAO (Δ%: − 23.67 ± 13.26, p < 0.001) levels compared to baseline.

**Table 3:** Parameters of inflammation and oxidative stress of the study population at baseline and after 28 days of the active phase of VLCKD

Parameters (N = 12)	Baseline	Day 28 of VLCKD	p-value	Δ %
Ox-LDL (μg/mL)	744.00 ± 307.54	56.75 ± 246.77	** < 0.001**	− 25.53 ± 7.64
dROMs (U Carr)	225.00 ± 119.70	177.81 ± 102.10	**0.001**	− 21.10 ± 12.86
TMAO (µM)	4.89 ± 1.40	3.79 ± 1.40	** < 0.001**	− 23.67 ± 13.26

A p-value in bold type denotes a significant difference (p < 0.05). *VLCKD* very low-calorie ketogenic diet; Δ%, percentage change, *ox-LDL* oxidized low-density lipoprotein, *dROMS*, reactive oxygen metabolites, *TMAO* trimethylamine n-oxide

Table 4 reports the simple correlations among changes in the Sartorius scores and changes in the study parameters after 28 days of the active phase of VLCKD. Changes in the Sartorius score positively correlated with changes in ox-LDL (p = 0.002), dROMs (p < 0.001), and TMAO (p < 0.001) levels, and negatively with PhA (p < 0.001).

**Table 4:** Simple correlations among the Sartorius scores and changes in the study parameters after 28 days of the active phase of VLCKD

Δ%	Δ% Sartorius score	
	Simple Correlation	
	r	p-value
Anthropometric parameters		
Weight (kg)	0.038	0.908
BMI (kg/m^2^)	0.035	0.914
WC (cm)	− 0.211	0.510
BIA parameters		
R (Ω)	0.312	0.323
Xc (Ω)	− 0.896	** < 0.001**
Na/K	− 0.149	0.643
BCM (kg)	− 0.839	**0.001**
TBW (%)	− 0.679	**0.015**
ECW (%)	0.973	** < 0.001**
ICW (%)	− 0.951	** < 0.001**
FM (kg)	0.607	**0.036**
FM (%)	0.627	**0.029**
FFM (kg)	− 0.691	**0.013**
FFM (%)	− 0.680	**0.015**
MM (kg)	− 0.711	**0.009**
MM (%)	− 0.707	**0.010**
BCMI (kg/m^2^)	− 0.243	0.447
PhA (°)	− 0.953	** < 0.001**
Biochemical parameters		
Fasting plasma glucose (mg/dl)	0.192	0.571
Insulin (μU/ml)	0.396	0.257
HoMA-IR	0.353	0.317
Total cholesterol (mg/dL)	− 0.266	0.429
HDL cholesterol (mg/dL)	− 0.129	0.706
LDL cholesterol (mg/dL)	− 0.149	0.662
Triglycerides (mg/dL)	− 0.281	0.402
Azotemia (mg/dL)	− 0.302	0.340
Creatinemia (mg/dL)	− 0.024	0.941
Uricemia (mg/dL)	− 0.291	0.358
AST (U/L)	0.519	0.084
ALT (U/L)	0.326	0.301
γGT (U/L)	0.020	0.951
ESR (mm/h)	0.314	0.410
Sodiemia (mmol/l)	0.152	0.637
Potassiema (mmol/l)	0.217	0.497
Dermatological parameter		
DLQI score	− 0.170	0.596
Inflammation and oxidative stress parameters		
Ox-LDL (μg/mL)	0.796	**0.002**
dROMs (U Carr)	0.953	** < 0.001**
TMAO (µM)	0.936	** < 0.001**

A p-value in bold type denotes a significant difference (p < 0.05). Δ%, percentage change; *BMI* body mass index, *WC* waist circumference, *BIA* body impedance analysis, *R* resistance, *Xc* reactance, *BCM* body cell mass, *TBW* total body water, *ECW* extracellular water, *ICW* intracellular water, *FM* fat mass, *FFM* fat free mass, *MM* muscle mass, *BCMI* body cell mass index, *PhA* phase angle, *HoMA-IR* homeostatic model for assessment of insulin resistance, *HDL* high density lipoprotein, *LDL*, low density lipoprotein, *AST*, aspartate transaminase, *ALT* alanine transaminase; γGT, γglutamyltransferase; ESR, erythrocyte sedimentation rate; DLQI, dermatology life quality index; ox-LDL, oxidized low-density lipoprotein; dROMS, reactive oxygen metabolites, *TMAO* trimethylamine n-oxide

## Discussion

In this prospective pilot study, a cohort of 12 women with HS and overweight or obesity underwent the active phase of VLCKD for 28 days. As expected, at the end of the active phase of VLCKD, anthropometric measurements showed significant reductions in both BMI and WC, and, for body composition, FM decreased significantly, while FFM showed a slight increase. This result was in line with a 12-week study on women with polycystic ovary syndrome (PCOS) undergoing a ketogenic diet, where the authors found significant weight loss primarily in FM [42]. In fact, despite a slight absolute decrease in FFM (kg), the key highlight was a substantial and statistically significant increase in its percentage value [42]. Merra et al. also demonstrated that, after 3 weeks of dietary intervention, VLCKD was effective in reducing body weight without inducing muscle mass loss, thus preventing the risk of sarcopenia [43]. In line with the current literature, this observation pointed out the importance of ensuring an adequate intake of protein, both in terms of quantity (1.2–1.5 g/kg ideal body weight) and quality, with high-biological-value protein preparations such as those we used in this study. Nutritional ketosis, as evidenced in rat hearts and diaphragms, inhibits the oxidation of branched-chain amino acids and reduces the release of the gluconeogenic amino acid alanine [44, 45]. Sherwin et al. observed decreased nitrogen excretion and hypoalaninemia in fasting men with BHB infusion, indicating potential anti-catabolic effects [46]. Koutnik et al. further support this notion, suggesting that nutritional ketosis may attenuate muscle protein breakdown, allowing for the maintenance or even gain of skeletal muscle mass despite lower insulin levels [47]. Finally, the preservation of muscle mass has been included among the benefits of ketogenic diets [48], due to the synergistic effects exerted by the reduction in visceral adipose tissue and obesity-related pro-inflammatory status and the modulation of the gut microbiota [49–51].

In addition, there were also notable increments observed in PhA, and this was consistent with previous research [22, 30, 31]. PhA is a BIA parameter that serves as an indicator of cellular health and the distribution of body fluids. It has been recognized as a prognostic marker for both the incidence of illnesses and the likelihood of mortality in cases of chronic inflammatory conditions [52]. It is worth noting that PhA values tend to be diminished in a significant portion of inflammatory disorders, which encompass conditions like psoriasis and HS [19, 53].

For the first time, our study demonstrated a significant decrease in the Sartorius score, index of HS severity, after 28 days of the active phase of VLCKD compared to baseline. Notably, this reduction coincided with significant decreases in TMAO, dROMs, and LDLox, markers indicative of dysbiosis, oxidative stress, and cardiovascular risk, respectively. Our correlation analysis further strengthened this association. Specifically, changes in the Sartorius score exhibited positive correlations with changes in TMAO, dROMs, and LDLox. These findings carry significant implications regarding the potential benefits of VLCKD, particularly for patients struggling with HS along with overweight or obesity, a category of patients particularly exposed to dysbiosis, oxidative stress, and a high risk of cardiovascular diseases [54–56]. In this regard, our prior study revealed heightened circulating levels of TMAO, a gut-derived metabolite linked to inflammation and cardiometabolic risk, in 35 patients with HS and with overweight or obesity compared to control subjects [14]. Remarkably, TMAO levels were correlated with the clinical severity of HS, and this correlation remained significant even after adjusting for common confounding factors [14]. In this context, the reduction of inflammation and oxidative stress (as evidenced by the reduction of ox-LDL and dROMs levels, respectively) and the improvement of intestinal dysbiosis (as represented by the reduction of TMAO levels) induced by VLCKD, taken together, may represent the pathophysiological mechanism that is associated with the beneficial effects of this dietary therapy in reducing the clinical severity of HS.

Current research explores how dietary choices can complement traditional treatments for inflammatory disorders, notably HS [57]. Patients with HS are increasingly interested in managing their condition through dietary adjustments, though scientific evidence for specific dietary interventions remains scarce. Anecdotal reports suggest strategies like eliminating dairy, reducing simple carbs, avoiding nightshade vegetables, and certain supplements may benefit some patients with HS [57]. During a VLCKD, there is an increase in the levels of ketone bodies. These compounds are believed to have the potential to counteract inflammation and provide antioxidant effects within the body [58]. This, in turn, could potentially reduce the occurrence of age-related diseases and viral infections like COVID-19 [59, 60]. However, the exact molecular mechanisms responsible for how ketone bodies alleviate oxidative stress and inflammation remain a topic of ongoing investigation. Recent research, drawing from preclinical and clinical studies, has put forth the idea that ketone bodies may induce a controlled level of stress in the mitochondria [58]. This stress, in a subsequent step, activates specific factors such as Nrf2, sirtuins 1 and 3, and AMP-activated kinases, thereby initiating an adaptive response. This adaptive response results in an anti-oxidative and anti-inflammatory state, an improved function of the mitochondria, and the activation of mechanisms for cell repair and regeneration [58].

Finally, we would like to emphasize some safety results that emerged from our study. Glucose and lipid profiles, electrolytes, uric acid, liver enzymes, and markers of kidney function were assessed at both baseline and upon completion of the active phase of VLCKD. Notably, by the end of this active phase, total cholesterol and LDL cholesterol showed significant reductions compared to baseline, indicating a transient enhancement in cardiovascular risk. As for the non-significant changes observed in the HoMA-IR index, despite the decreased carbohydrate intake and weight loss, these are likely attributable to the short treatment duration, which may not allow for substantial improvements in glucose metabolism to manifest. Nevertheless, the absence of significant changes in biochemical parameters such as sodium, potassium, uric acid, and creatinine is an encouraging sign of the VLCKD's safety in patients with HS.

In the present study, it is essential to recognize certain limitations: (a) The sample was limited, and this may have affected the generalizability of the results. However, HS is a relatively rare disease with an overall incidence of approximately 0.00033–4.1% [61]; (b) Lack of a control group. However, for the short treatment period, a comparison with another diet, such as the Mediterranean diet, would have been ineffective, requiring a longer period for comparable results; (c) The sample included only women, limiting consideration of the effect of VLCKD on men. However, HS afflicts women more, and our results could be better applied based on this sex disproportion [62]; (d) A comprehensive analysis of safety and interactions with drugs used to treat HS has not been conducted. Assessing these interactions will be crucial to ensuring the safety of the treatment; (e) Furthermore, this prospective pilot study has the sole purpose of reporting the efficacy of VLCKD in women with HS and not its superiority over other dietary treatments. Further studies, preferably randomized, are needed to compare the efficacy and safety of VLCKD with other dietary approaches available for patients with HS.

We also outline the strengths of the study: (a) VLCKD used highly controlled replacement meals, ensuring a strictly monitored caloric and nutritional intake. This contributed to maintaining a highly controlled and standardized diet for all participants; (b) The patients were followed by a specialized multidisciplinary team that continuously monitored adherence to VLCKD. Ultimately, the results can be used to inform future studies, that is the real strength of this study.

## Conclusion

This prospective pilot study is the first evidence of the efficacy and safety of a VLCKD in reducing the clinical severity of HS only after 28 days of dietary therapy. Taken together, our data suggest that a VLCKD can be considered a successful dietary strategy and a safe therapeutic option for the management of women with HS. The main effects depend on the reduction of inflammation and oxidation and the improvement of the intestinal microbiota, which, together, alter the inflammatory and oxidative environment typical of this skin disease.

### Supplementary Information

**Additional file 1.** Example of VLCKD diet therapy with meal replacement.

## Data Availability

The datasets used and/or analyzed during the current study are available from the corresponding author on reasonable request.
