# Supplementary material for: Very low-calorie ketogenic diet (VLCKD) in the management of hidradenitis suppurativa (Acne Inversa): an effective and safe tool for improvement of the clinical severity of disease. Results of a pilot study
Source: J Transl Med. 2024 Feb 13;22:149. doi: 10.1186/s12967-024-04853-0 (PMC10863195; doi:10.1186/s12967-024-04853-0)
Supplement: Supplementary file 1 — Additional file 1. Example of VLCKD diet therapy with meal replacement. [file 12967_2024_4853_MOESM1_ESM.docx]

**Example of VLCKD diet therapy with meal replacement**

**(New Penta Srl, Cuneo, Italy)**

**Breakfast (meal replacement, cappuccino drink)***

| AVERAGE NUTRITIONAL ANALYSIS | |
| --- | --- |
| Kcal/KJ | 91 / 386 |
| Fat (g) | 0,5 |
| *of which saturated fatty acids (g)* | 0,4 |
| Carbohydrates (g) | 2,7 |
| *of which sugars (g)* | 1,7 |
| Fiber (g) | 1,7 |
| Protein (g) | 18 |
| Salt (g) | 0,33 |

**Mid-morning snack (meal replacement, milkshake-flavored drinks)***

| AVERAGE NUTRITIONAL ANALYSIS | |
| --- | --- |
| Kcal/KJ | 88 / 371 |
| Fat (g) | 0,7 |
| *of which saturated fatty acids (g)* | 0,2 |
| Carbohydrates (g) | 1,4 |
| *of which sugars (g)* | 1,1 |
| Fiber (g) | 2,5 |
| Protein (g) | 18 |
| Salt (g) | 0,32 |

**Lunch, meal replacement (protein pasta)***

| AVERAGE NUTRITIONAL ANALYSIS | |
| --- | --- |
| Kcal/KJ | 143 / 605 |
| Fat (g) | 1,3 |
| *of which saturated fatty acids (g)* | 0,9 |
| Carbohydrates (g) | 1,6 |
| *of which sugars (g)* | 1,2 |
| Fiber (g) | 3,2 |
| Protein (g) | 15 |
| Salt (g) | 0,27 |


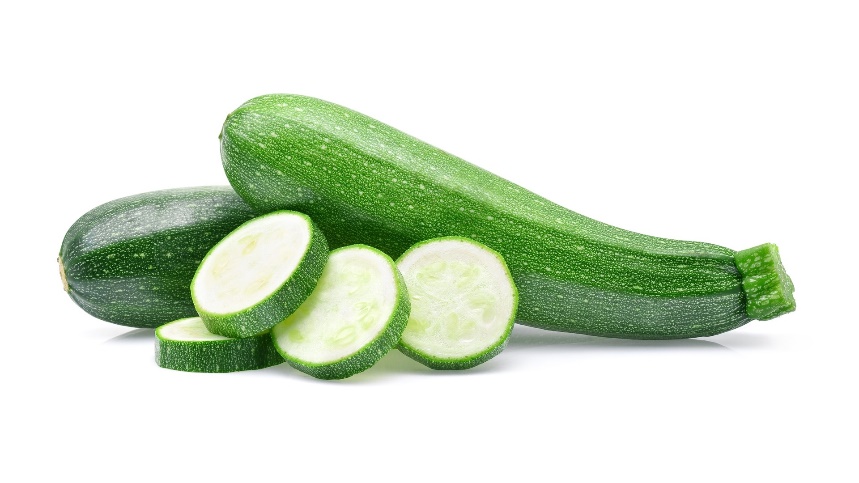


**+ 200 g of low glycaemic index vegetables (e.g. courgettes)**


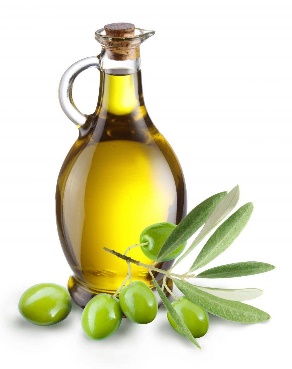


**+ 15 g of extra virgin olive oil**

**Dinner, meal replacement (type omelette)***

| AVERAGE NUTRITIONAL ANALYSIS | |
| --- | --- |
| Kcal/KJ | 113 / 476 |
| Fat (g) | 2,6 |
| *of which saturated fatty acids (g)* | 0,1 |
| Carbohydrates (g) | 2,5 |
| *of which sugars (g)* | 0,5 |
| Fiber (g) | 0,5 |
| Protein (g) | 20 |
| Salt (g) | 1,4 |


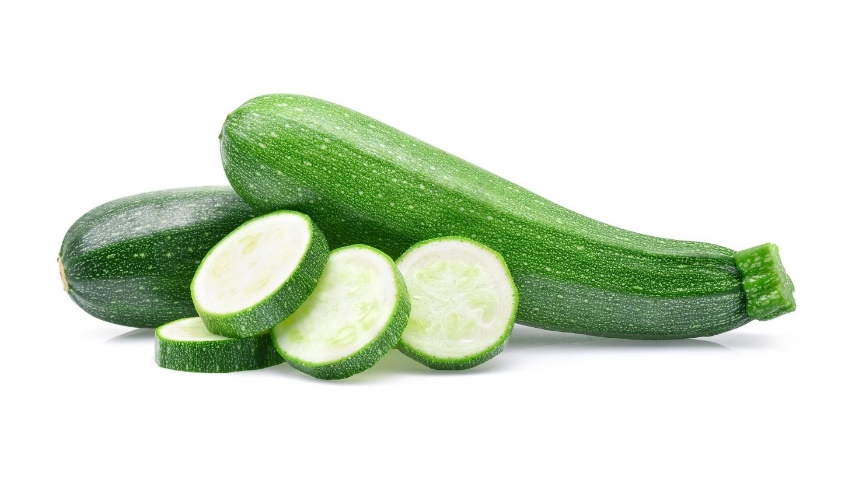


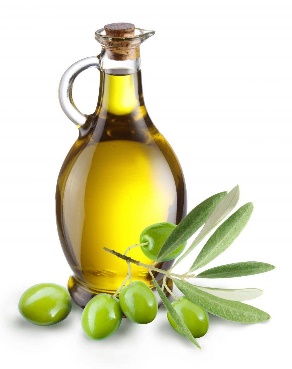
**+ 200 g of low glycaemic index vegetables (e.g. courgettes)**

**+ 15 g of extra virgin olive oil**


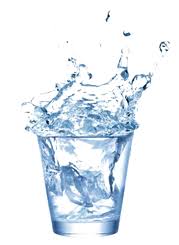
**In addition:**

**+ Drink at least 2.5 liters of water *per* day**

**+ Multivitamin and saline supplements to maintain physiological acid/base balance (PentaCal, New Penta, Srl., Cuneo, Italy) (B complex vitamins, C and E vitamins, minerals, including potassium, sodium, magnesium, calcium, and omega-3 fatty acids (250 mg/day for eicosapentaenoic acid –EPA- and docosahexaenoic acid –DHA-).**

| **Calories and nutrients from meal replacements** | |
| --- | --- |
| Kcal/KJ | 435/1838 |
| Fat (g) | 5,1 |
| *of which saturated fatty acids (g)* | 1,6 |
| Carbohydrates (g) | 8,2 |
| *of which sugars (g)* | 4,5 |
| Fiber (g) | 7,9 |
| Protein (g) | 71 |
| Salt (g) | 2,32 |

| **Calories and nutrients from EVOO and vegetables** | | |
| --- | --- | --- |
|  | EVOO  (30 g) | Vegetables (e.g. courgettes)  (200 g + 200 g) |
| Kcal/KJ | 269,7/1108,8 | 44/192 |
| Fat (g) | 29,97 | 0,4 |
| *of which saturated fatty acids (g)* | 0 | 0 |
| Carbohydrates (g) | 0 | 5,6 |
| *of which sugars (g)* | 0 | 0 |
| Fiber (g) | 0 | 5,2 |
| Protein (g) | 0 | 5,2 |
| Salt (g) | 0 | 4 |

| Average values of the VLCKD | |
| --- | --- |
| Kcal/KJ | 748,7/3138,8 |
| Fat (g) | 35,47 |
| *of which saturated fatty acids (g)* | 1,6 |
| Carbohydrates (g) | 13,8 |
| *of which sugars (g)* | 4,5 |
| Fiber (g) | 7,9 |
| Protein (g) | 76,2 |
| Salt (g) | 6,32 |

*** https://pentadiet.it/prodotti**
